# Moderate hypofractionation for prostate cancer

**DOI:** 10.18632/oncotarget.21386

**Published:** 2017-09-29

**Authors:** Stefano Arcangeli, Giorgio Arcangeli

**Affiliations:** Stefano Arcangeli: Department of Radiation Oncology, San Camillo-Forlanini Hospitals, Rome, Italy

**Keywords:** prostate cancer, hypofractionated radiotherapy

At the end of the last century, Brenner and Hall [1] argued that the fractionation sensitivity of prostate cancer was different from most tumors and normal tissues, with the opportunity to increase the therapeutic ratio by treating this cancer with hypofractionation (fewer, larger daily fractions). Nearly two decades later, the use of moderate hypofractionation for prostate cancer has become an established strategy, supported by international guidelines [i.e: NCCN Guidelines^®^, Version 2.2017]. The stringent data are provided by three large randomized non-inferiority trials [2-4], which demonstrated that a shorter schedule remains beneficial (in terms of 5-years relapse-free survival) whilst decreasing treatment duration, with clear socioeconomic benefits for both patients and the health care. As it is inferred from the HRs for the primary endpoint — all consistently below 1 — the outcomes were the same irrespective of the inclusion of patients with different risk groupings, use of androgen deprivation therapy and different radiotherapy regimens. Accordingly, hypofractionation seems effective for most men with prostate cancer, and is entitled to become the preferred approach. With respect to treatment-related toxicity, no significant differences in grade ≥2 late GI and GU toxicity was observed between the hypofractionated and the conventional arm in two of the trials where the same experimental schedule (60 Gy in 3 Gy daily fractions) was adopted [3, 4]; conversely, the increased rate of late adverse events reported in two other large studies [2, 5] should be likely attributed to the biologically higher doses employed in the hypofractionated arm of both trials. Should these findings lead to abandonment of the 8-9 week standard for all patients with prostate cancer? Some concerns remains about the relatively short lenght of follow up, as well as the substantial proportion of patients with low and intermediate risk included in the aforementioned trials [2-4]. In view of the potential risks of a late radiation-induced toxicity, many clinicians are still reluctant to accept the widely adoption of hypofractionation in the daily practice. Additionally, non-threatening cancers would likely relapse later in the course of follow-up, owing to the presumably slower tumor growth. Taken together, these observations might indicate that a longer follow-up is required to show more reliable outcomes. This relative lack of long-term data can be filled, to some extent, by the recent study that we co-authored [6], in which final results with 9 years of follow-up are showed. In keeping with other superiority trials [7, 8], we concluded that moderately hypofractionated radiotherapy, consisting in 3.1 Gy x 20 fractions — very close to that used in two of the non-inferiority trials [3, 4] — did not result in lower rates of late complications (the primary endpoint), nor in improved efficacy. However, it may add some insightful suggestions to the existing knowledge: first, this is the phase III study on moderate hypofractionation for prostate cancer with the longest follow-up. Although it was not powered to show differences in tumor outcomes, a trend in favor of hypofractionation in the actuarial biochemical free-survival was found. Additionally, a postrandomization analysis revealed an association between the use of hypofractionation and a decreased risk of death from prostate cancer, thus supporting the value of hypofractionation also when a robust outcome measure is considered. This hypothesis is further strenghtened by the solely inclusion of patients with high-risk tumors (and all receiving androgen deprivation therapy) in this trial. Finally, long-term toxicity paralleled the previously published 3-years data, with no significant difference in G2 or worse adverse effects between the two treatment arms. Noteworthy, a minor (G1) late GU toxicity, namely: macroscopic hematuria, was significantly more frequent in the hypofractionated arm (16.5% v 3.6%, P =.009), and can be ascribed to the use of a nowadays obsolete 3D conformal technique. In conclusion, accumulating evidence seems to show that a shortened course of radiotherapy is as safe and effective as a long course of conventionally fractionated radiotherapy, at least at early time-points, thus supporting current practice-changing trends. Long-term data from large non-inferiority trials are mandatory to confirm this strategy, but will probably have a limited effect on clinical practice by the time they will be published. Indeed, several trials of stereotactic body radiation therapy (SBRT) have been already concluded, holding sufficient promises that it might become the procedure of choice for the management of organ-confined prostate cancer, especially in light of the increasing number of elderly patients in need for treatment.
